# Bibliometric Analysis on Supercritical CO_2_ Power Cycles for Concentrating Solar Power Applications

**DOI:** 10.3390/e23101289

**Published:** 2021-09-30

**Authors:** Miguel Angel Reyes-Belmonte, Rafael Guédez, Maria José Montes

**Affiliations:** 1 Department of Chemical and Energy Technology, School of Experimental Sciences and Technology (ESCET), Rey Juan Carlos University, 28933 Madrid, Spain; 2 Department of Energy Technology, KTH Royal Institute of Technology, Brinellvägen 68, 100 44 Stockholm, Sweden; rafael.guedez@energy.kth.se; 3 E.T.S. Ingenieros Industriales-UNED, C/Juan del Rosal 12, 28040 Madrid, Spain; mjmontes@ind.uned.es

**Keywords:** sCO_2_, supercritical CO_2_, supercritical fluids, CSP, concentrating solar power, solar energy, power cycles, bibliometric, scientometrics

## Abstract

In recent years, supercritical CO_2_ power cycles have received a large amount of interest due to their exceptional theoretical conversion efficiency above 50%, which is leading a revolution in power cycle research. Furthermore, this high efficiency can be achieved at a moderate temperature level, thus suiting concentrating solar power (CSP) applications, which are seen as a core business within supercritical technologies. In this context, numerous studies have been published, creating the need for a thorough analysis to identify research areas of interest and the main researchers in the field. In this work, a bibliometric analysis of supercritical CO_2_ for CSP applications was undertaken considering all indexed publications within the Web of Science between 1990 and 2020. The main researchers and areas of interest were identified through network mapping and text mining techniques, thus providing the reader with an unbiased overview of sCO_2_ research activities. The results of the review were compared with the most recent research projects and programs on sCO_2_ for CSP applications. It was found that popular research areas in this topic are related to optimization and thermodynamics analysis, which reflects the significance of power cycle configuration and working conditions. Growing interest in medium temperature applications and the design of sCO_2_ heat exchangers was also identified through density visualization maps and confirmed by a review of research projects.

## 1. Introduction

The use of supercritical carbon dioxide (sCO_2_) as a working fluid for electricity generation systems, based on fossil fuel, nuclear power, or concentrating solar power (CSP), offers several advantages compared to other conventional schemes [1,2,3]. For nuclear or fossil energy, sCO_2_ is employed in the power cycle, yielding different supercritical Brayton layouts. In the case of CSP, sCO_2_ can perform as the working fluid in the power block, the heat transfer fluid (HTF) in the solar field, and/or in the thermal storage system. Thus, this introduction analyzes these possibilities, in addition to their integration in different schemes of solar thermal power plants (STPPs).

According to IRENA, the amount of globally installed CSP power has significantly increased since 2010, translating into a reduction in the levelized cost of electricity (LCoE) from 0.346 USD/kWh_e_ to 0.182 USD/kWh_e_ [4]. Nevertheless, this cost is still far from the 0.06 USD/kWh_e_ target established by the SunShot Initiative from the US Department of Energy (DOE) [5]. In addition, it is important to note that the decrease in LCoE during the past decade has been mainly motivated by a reduction in the solar field cost (which represents 40% of the STPP investment cost), due to a greater economy of scale. Although the LCoE can be reduced by lowering costs, a second approach to improving CSP competitiveness is via increasing the global thermal performance of the STPPs. This is the pathway established by the Gen3 CSP Roadmap [6] and the Australian Solar Thermal Research Initiative (ASTRI) [7], in which the use of sCO_2_ is a key driver.

In the following sections, the current state of STPP subsystems employing sCO_2_ are reviewed. First, sCO_2_ power cycle layouts that are best integrated into CSP are described, second, sCO_2_ solar receivers are reviewed, and finally, integration schemes and thermal energy storage systems are proposed.

### 1.1. Supercritical CO_2_ Brayton Cycles 

Supercritical CO_2_ power cycles based on a closed recompression layout present higher thermal efficiency than those of superheated or supercritical steam Rankine cycles at the temperature range of STPPs. This high efficiency is based on the peculiar thermophysical properties of CO_2_ in the region near the critical point (7.38 MPa, 31 °C). The sCO_2_ density close to and above the critical pressure is extremely high, so the compressor power is reduced [8]. This also involves reducing the turbine inlet temperature for the same thermal efficiency, and global conversion efficiencies from 40% to above 50% can be achieved for turbine inlet temperatures of 500 and 700 °C, respectively [9,10].

In addition, sCO_2_ cycles exhibit other technological advantages compared to conventional steam Rankine cycles. For example, the turbomachinery size is smaller when operating near to the critical point, which implies good operational flexibility and the possibility of a lower LCoE; in addition, the sCO_2_ is less corrosive than steam at high temperature. Significant technological challenges also apply, including development of components capable of withstanding the demanding supercritical working conditions, such as the design of the primary heat exchanger needed when coupling the solar field and the power block for indirect configurations. Another challenge is the impact of the compressor inlet temperature on the power cycle efficiency [11]. When the ambient temperature becomes higher than the cooler design conditions, the power cycle efficiency can be significantly penalized. Thus, it is a challenge to integrate sCO_2_ power cycles into the STPPs, particularly for dry configurations [12,13]. To address this limitation, some researchers have proposed a modified working fluid whereby CO_2_ is blended with certain additives to enable condensation at higher ambient temperatures, enabling required peak temperatures to be withstood without penalizing the power cycle efficiency, thus resulting in large reductions in LCoE [14].

Although sCO_2_ cycles were mainly developed for nuclear applications, a growing interest in the integration of sCO_2_ into STPPs has recently arisen. Turchi et al. [15] presented a supercritical STPP scheme based on modular towers and a conventional recompression supercritical layout. From the perspective of the power cycle, this configuration does not present new features; however, it is discussed below because it presents a complete integration scheme in a supercritical plant. In a later work, Neises and Turchi [16] undertook detailed analysis of the partial cooling and the recompression configurations, concluding that the partial cooling configuration offers important advantages for CSP applications, such as a large temperature difference in the primary heat exchanger, which implies a smaller size of the solar receiver and higher efficiency. Finally, in [17], sCO_2_ turbine efficiency at the scale of operational CSP projects was assessed to promote this technology at commercial levels.

Subsequently, two important review works were published. In [2], a general evaluation of sCO_2_ cycles for power generation was presented. Wang et al. [18] identified and analyzed six possible supercritical layouts that can be indirectly coupled to a molten salts central receiver, i.e., simple recovery cycle, recompression cycle, precompression cycle, intercooling cycle, partial-cooling cycle, and split expansion cycle. This analysis identified different parameters for the comparison, and highlighted the thermal efficiency; the complexity of the cycle compared to the simplest (i.e., the recompression cycle); and the temperature difference of the sCO_2_ in the primary heat exchanger which, as seen above, determines the molten salt temperatures and can result in lower investment in the coupled solar subsystem. This study concludes that no one layout is better than the others. The final choice depends on the specific operating and ambient conditions, and should account for the annual performance of the STPP.

A later work of NREL [19] analyzed two sCO_2_ cycles—the recompression and partial-cooling cycles—based on the global STPP performance. The authors concluded that the partial-cooling cycle has lower investment costs and generates more net electricity based on the larger temperature difference in the primary heat exchanger.

Finally, it is important to note that one of the key elements for the feasibility of this technology is that the design of the primary heat exchanger connects the solar field and the power block because, in general, the fluids are not the same (indirect integration schemes). This is the case of a molten salt central receiver coupled to an sCO_2_ layout. Several designs have been proposed in the literature for molten salt-to-CO_2_ heat exchangers, for both nuclear and CSP applications [20,21,22,23], and sCO_2_-to-liquid sodium compact heat exchangers for sodium-cooled fast reactors [24]. The simplest design is the Shell and Tube Heat Exchanger (STHX), in which the molten salt flows through the shell while the sCO_2_ circulates through the tubes. For this type of heat exchanger, a new sCO_2_ layout is proposed in [25], in which the primary thermal energy is supplied through the low-pressure side of the layout, downstream of the turbine (approximately 85 bar). Different proposals for particle-to-sCO_2_ heat exchangers include using a moving packed-bed [26], fluidized-bed [27,28], or shell-and-plate heat exchanger [29,30].

### 1.2. Supercritical CO_2_ Solar Receivers

As in the case of the supercritical CO_2_ cycles for CSP, the research on sCO_2_ solar receivers is relatively new, although there now appears to be a growing interest. The review presented in this section is focused on sCO_2_ solar central receivers (CRs). As noted in [15], due to the high pressure required for sCO_2_, its application to parabolic trough (PT) fields is difficult, although theoretical studies have been conducted [31].

A previous study reviewed compact heat exchanger (CHE) structures and the possibility of integrating them in pressurized solar receivers [32]. Although the authors claimed that their work may be the starting point for further research, few additional studies have been based on their conclusions, as discussed below.

One of the first supercritical CO_2_ central receivers proposed was based on the external tubular receiver concept [33,34]. This design is intended to heat the air to 800 °C with a pressure of 5–7 bar; however, the adaption of this receiver to enable direct coupling to a sCO_2_ power cycle working at 200 bar and 700 °C has also been considered. In this case, additional requirements would be necessary to withstand the high pressure and temperature, and to enhance the heat transfer to the supercritical phase.

In the study presented in [35], the CHE concept was used in a 3 MW_th_ cavity receiver for sCO_2_. This receiver consists of several plates joined by diffusion, with rectangular fins between them, in such a manner that square-shaped channels are formed. The optimal geometry of this CHE structure was selected through an optimization process, as explained in the same work.

Another interesting configuration was proposed in [36]. In this case, an intermediate fluid, i.e., pressurized air, directly receives the radiation, affecting a cavity receiver provided with a quartz window and a porous structure. This working fluid transfers its thermal energy to the sCO_2_ that circulates through ducts embedded in the porous matrix itself.

Finally, a recent work by the National Renewable Energy Laboratory (NREL) presented two concepts for sCO_2_ central receiver designs [37]. The first is a cavity receiver for a 2 MW_e_ power cycle, and the second is a surround external receiver for a 10 MW_e_ cycle. In both designs, the sCO_2_ circulates through a compact structure consisting of two attached plates with a wavy fin structure between them, which acts as the absorber surface for the concentrated solar radiation. The main difference is that the absorber plates are arranged to form a cavity in the first case, whereas they are arranged to form an external cylindrical receiver in the second. Because radiation losses would be very high in this last case, a radiation trap was designed, consisting of small quartz cylinders perpendicular to the wall, which reduce radiation and convection losses. In this manner, the receiver thermal efficiency remains high (80%) when working at temperatures of approximately 750 °C. In both designs, the objective of 0.06 USD/kWh_e_ established by the SunShot Initiative is attained.

### 1.3. Integration Schemes for sCO_2_ STPPs

Most supercritical STPP layouts use indirect coupling because the HTF in the solar field and the working fluid in the power cycle are different. This is the configuration selected by the above-mentioned Gen3 CSP Roadmap [6] and ASTRI programs [7], in which the power cycle is a sCO_2_ cycle, and different schemes are defined depending on the HTF in the central receiver: liquid sodium [38] or molten salt, and solid (single-phase particles) or gas. In these schemes, it is necessary to incorporate a primary heat exchanger between both subsystems. The design of this heat exchanger is a key issue for the technological feasibility of these plants, and several proposals are found in the literature, for reducing both particles-to-sCO_2_ and molten salt-to-sCO_2_ [22,23,39]. A brief description of each of the above schemes is given in the following paragraphs.

The molten salt receiver scheme coupled to a sCO_2_ cycle is the most conventional approach, and there are several works in the literature about this configuration. In this scheme, the molten salts also perform as the thermal storage fluid, and the proposed configuration is usually a direct two-tank TES, although a thermocline can also be used [18,23,40]. To achieve the objectives of the SunShot and ASTRI programs, it is necessary to work at a higher temperature than achieved at commercial STPPs; the HTF temperature at the outlet of the solar receiver should reach 700 °C, which also implies the use of advanced ternary salts [41].

An integration scheme in which a liquid metal solar receiver is coupled to a sCO_2_ cycle is analyzed in [42], where a tubular sodium receiver, high-temperature phase-change material (PCM) storage system, and sCO_2_ power block are considered.

The STPP based on a particle receiver coupled to a sCO_2_ cycle is represented in few works in the technical literature, although a global integration scheme is presented in [6,34], and several models have been developed for the falling particle receiver [43], the bladed particles receiver [44], the thermal storage system in particles [45], and the primary heat exchanger between the solar field and the power block [39].

Regarding the pressurized air receiver coupled to a sCO_2_ cycle, the works of Li et al. [46] and Trevisan et al. [47] can be highlighted. A design and simulation model of a sensible-packed bed thermocline (PBT) for pressurized air was proposed in both works.

All the schemes described above match the indirect coupling. To conclude this section, we discuss the direct integration schemes between the solar field and the power block where sCO_2_ is used both as the HTF and working fluid. Turchi et al. [15] presented a scheme for a supercritical STPP based on modular towers. Each modular tower is provided by its sCO_2_ power block, and, because of the turbine/compressor compactness, it is possible to allocate them in the tower. As a result, the piping is reduced, thus also decreasing the pressure and heat loss, and improving the transient response.

Although most of these direct integration schemes are intended to be coupled to indirect thermal storage in molten salts [15], it should be noted that the cascaded PCM storage system, proposed in [48], is specifically targeted at the efficient operation of high-temperature sCO_2_ cycles. Other studies proposed a direct coupling using a thermocline system. In this manner, Kelly et al. [49] presented a thermocline system based on a matrix of individual vessels with reduced dimensions to avoid a large wall thickness. A more theoretical model of the charge/discharge operation was presented in [50].

To summarize this introduction, the use of sCO_2_ in CSP is a recent but promising topic of investigation that is currently supported by several research programs. The number of research areas and new proposals has grown rapidly in recent years, aimed at developing more efficient and competitive STPPs. As a result, it is a challenge to undertake a review of the existing literature on supercritical CO_2_ power cycles for CSP applications. Bibliometrics tools can provide insights into the main researchers and institutions engaged in the topic, and the manner in which they are connected. This approach can also identify the main research trends and popular research topics that provided the motivation for this review article [51].

Table 1 presents similar bibliometric analyses related to power cycle technologies and concentrating solar power applications. As can be observed, two bibliometric research works have been recently published about supercritical CO_2_ power cycles [52,53]. However, both of these works covered the topic from a general perspective rather than analyzing the potential of the technology when coupled to concentrating solar power applications. In addition, it can be noted that most of the existing bibliometric studies reviewed the literature related to power cycles or solar energy, but not the combined application of both technologies.

Despite recent interest in supercritical CO_2_ for power generation, to the best of the authors’ knowledge, there are no specifical bibliometric studies regarding CSP applications from a global approach, which represents the novelty of this work. The objective of this study was to evaluate sCO_2_-CSP global research trends quantitatively and qualitatively through bibliometric techniques. The study’s conclusions will not only provide a better understanding of popular sCO_2_-CSP research areas, but may also influence scholars’ and scientists’ future research. To succeed in this ambitious enterprise, this paper is organized as follows: in the next section, the working methodology is presented; Section 3 discusses bibliometric indicators; Section 4 applies text mining techniques to identify research trends; and the project discussion in Section 5 connects current research trends and future topics for sCO_2_ in CSP.

## 2. Materials and Methods

Table 2 summarizes the number of supercritical CO_2_ related publications that were retrieved based on different question queries and consulted databases. Both Web of Science (WOS) and Scopus databases were consulted and publications including each query (whether regarding publication title, abstract, keywords, or KeywordPlus^®^) were retrieved. To account for most publications within the field, different expressions and search combinations were considered and the logical operator “or” was introduced to combine all of them, thus resulting in the total corpus data of the study. It can be seen that the number of indexed publications relating to sCO_2_ power cycles that also included the keyword “solar” was similar among Web of Science (441 publications) and Scopus (468) databases. In both cases, those publications accounted for one-third of total sCO_2_ power cycle publications, which indicates the relevance of CSP applications within sCO_2_ technologies.

The data set retrieved from the Web of Science (WOS) was preferred because it is claimed to contain journals with higher impact [63] and no previous studies covered sCO_2_ power cycles using this database [52,53]. Under that assumption, corpus data comprised 441 WOS indexed publications whose metadata (including full record and cited references) were exported for processing and network mapping visualization using the VOSviewer 1.6.16 software tool [64,65].

## 3. Results

In this section, several bibliometric indicators are presented and discussed to analyze the main researchers in sCO_2_ research with a focus on CSP applications, and to provide insights into technology trends.

**Figure 1:**
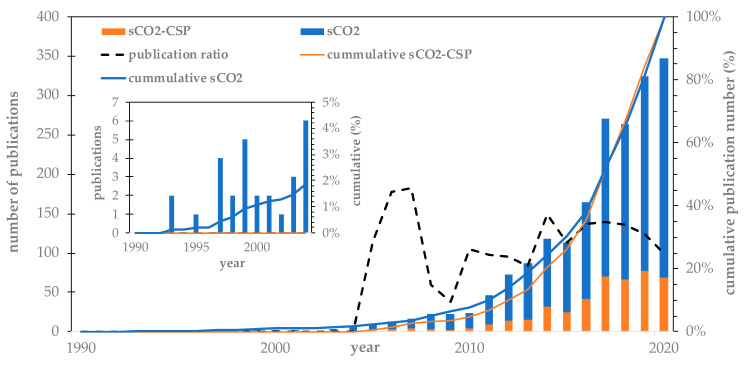
Publication evolution in sCO_2_ for CSP and sCO_2_.

Figure 1 shows the publishing evolution of sCO_2_ power cycle publications (sCO_2_) between 1990 and 2020 according to the WOS. As shown, the first WOS sCO_2_ power cycle publication was indexed in 1993, but the first publication related to CSP applications appeared in 2005. Subsequently, the relevance of sCO_2_ solar-related publications has continued to grow and now accounts for one-third of the annual sCO_2_ publications. Furthermore, 70% of the total number of publications were published after 2015. It can also be noted that the contribution of solar-related publications to the existing sCO_2_ literature is around 30%. During 2020, the number of sCO_2_ publications reached its maximum despite the slight decrease in solar-related publications compared to previous years.

**Figure 2:**
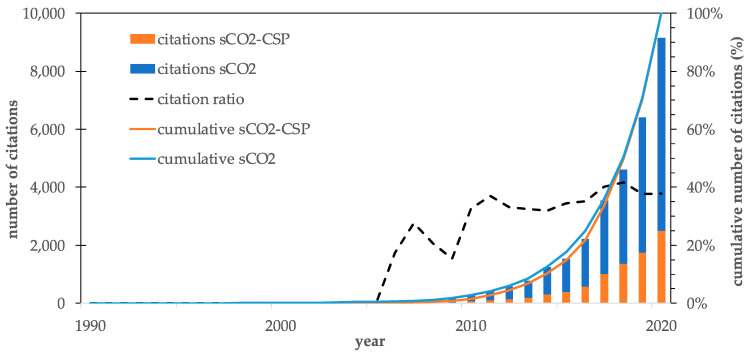
Citations’ evolution in general sCO_2_ publications and for CSP applications.

In terms of the number of citations, the contribution of sCO_2_-CSP-related publications is slightly higher compared to the publication ratio shown in Figure 1 because it accounts for almost 40% of the total citations, which indicates the growing relevance of CSP applications for sCO_2_ technologies. It can also be observed in Figure 2 that 80% of sCO_2_ power cycle citations were received after 2016.

### 3.1. Main Publishing Countries

**Figure 3:**
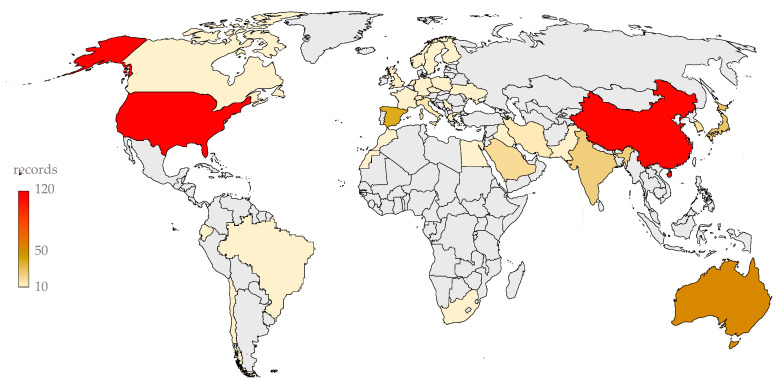
Publishing distribution for the topic of sCO_2_ for CSP applications (cumulative distribution until 2020).

As shown in Figure 3, the most productive countries in terms of WOS-indexed publications are the United States and China, which combined account for 43% of all sCO_2_-CSP documents.

Table 3 shows that the 10 most productive countries in sCO_2_-CSP account for 82.6% of publications in cumulative terms. A closer look at the scientific production during 2020 indicates a clear growth in Chinese and Spanish publications, and the cumulative production of the 10 most productive countries increased slightly, to 86.5% of the annual publications.

**Figure 4:**
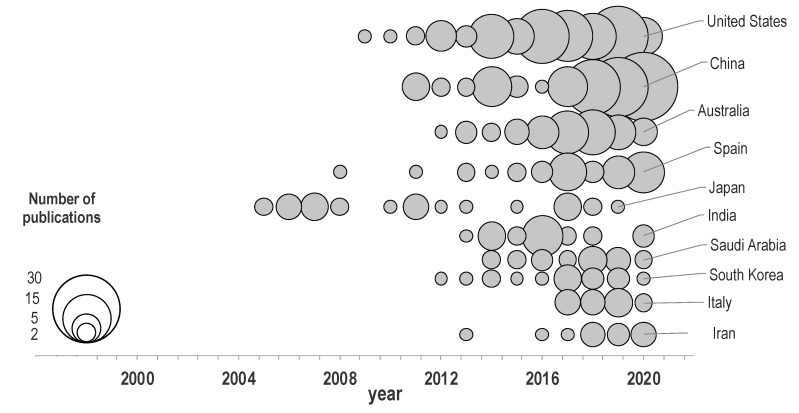
Publishing evolution of the most productive countries.

Figure 4 shows a clearer picture regarding the most productive countries in terms of publishing evolution. As shown, Chinese production has grown quickly during the past 3 years, whereas the production of Japan has decreased gradually, despite being the most productive country before 2010. The growing relevance of Italy, Iran, and Saudi Arabia in recent years can also be observed.

### 3.2. Main Publishing Institutions

Table 4 shows the most productive organizations regarding the number of indexed publications on sCO_2_ power cycles for concentrating solar power applications. Research institutions are ranked according to the number of publications. The number of authors that have published in the sCO_2_-CSP topic under the organization affiliation is reported, in addition to the accumulated number of citations (including self-citations). The publishing ratio (PC ratio) is determined as the ratio between the number of citations and publications for a given organization. The h-index of the institution is also provided considering only the number of publications and citations for the analyzed topic [66]. As shown, the ten most productive organizations are consistent with the most productive countries, with a clear dominance of United States which has four institutions in the top 10 rankings (United States Department of Energy, Sandia National Laboratory, National Renewable Energy Laboratory, and State University System of Florida). Regarding the number of citations received by the total publications, higher PC ratios are found for Xi’an Jiaotong University (China) and Doshisha University (Japan).

### 3.3. Main Publishing Authors

Table 5 gathers the 10 most productive authors in sCO_2_-CSP topics in terms of the number of publications. It also shows the number of citations received in this topic, the PC ratio, and the equivalent h-index considering only sCO_2_-CSP publications. The authors with the most common affiliations of those publications are also shown. As shown, most of the relevant authors belong to the most productive organizations (shown in Table 4) and most productive countries (gathered in Table 3), with the exceptions of Zhang, XR. from Peking University who exhibits the highest PC ratio, and Liu, M. from University of South Australia and Sanchez, D.; the latter two each have 10 research publications on sCO_2_-CSP. It can be observed that the most productive authors are located in Australia and United States, which is consistent with the location of large funding schemes, such as the SunShot and ASTRI initiatives [7].

### 3.4. Most Cited Publications in sCO_2_-CSP 

Table 6 shows the most cited publications in sCO_2_-CSP topics, with the publishing source, first author, country, and year of publication. Other relevant indicators, such as the total number of citations and the average citations per year, are included for comparison purposes. It is relevant that the most cited publications on this topic are recent review papers, which translates into a high average number of citations per year and indicates the research significance of sCO_2_-CSP topics. This is also supported by numerous research projects, as discussed in Section 4.2.

As shown, all publications are associated with the most productive countries and institutions, with the exception of the most cited publication from Korea Advanced Institute [1] and two publications from the University of Seville [2,70]. Despite the recent publication of these studies, their number of citations exceeds 50 per year.

### 3.5. Publication Distribution by Publishing Source

**Figure 5:**
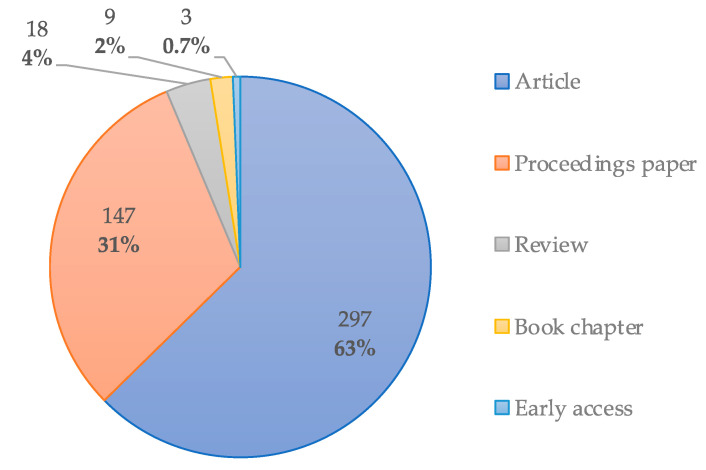
Document type distribution for sCO_2_-CSP publications.

Regarding the document type distribution, Figure 5 shows that most sCO_2_-CSP publications are articles (63%) followed by proceedings papers (31%), with the two categories combined accounting for 94% of corpus data.

**Figure 6:**
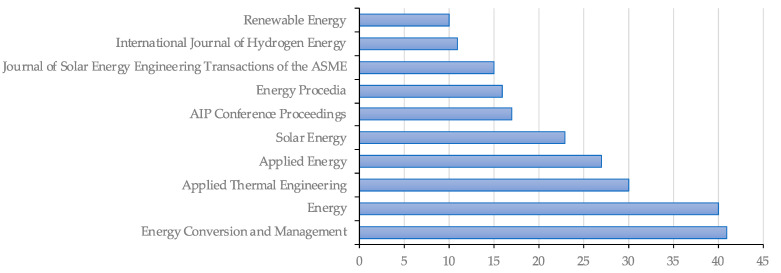
Most relevant publishing sources for sCO_2_ CSP publications.

Within article publications, the most relevant publishing sources for sCO_2_-CSP are *Energy Conversion and Management* and *Energy* journals, with 40 publications each, followed by *Applied Thermal Engineering* with 30, as shown in Figure 6. Among the 10 most common publishing sources for sCO_2_-CSP publications, it can be seen that dedicated solar-related sources, such as *Solar Energy* and the *Journal of Solar Energy Engineering Transactions of the ASME*. The figure also shows the sources for proceedings papers, such as those of the SolarPaces conference, which were published in *Energy Procedia* until 2014 and have been collected under *AIP Conference Proceedings* since the 2015 edition.

**Figure 7:**
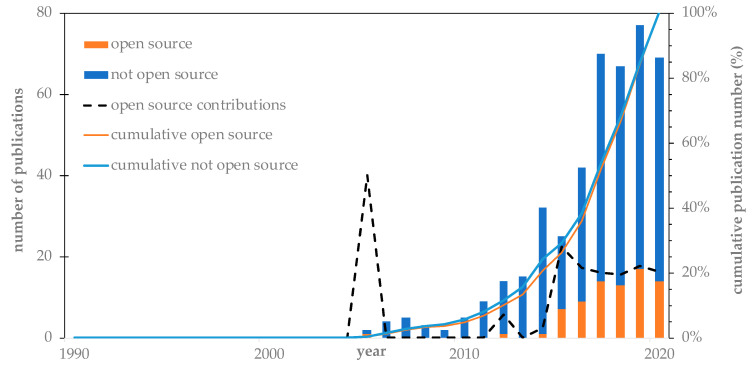
Publication source of sCO_2_-CSP publications.

Figure 7 shows the contribution of open-access publications within the existing sCO_2_-CSP literature. As indicated by the cumulative values, both sources followed the same trend in terms of publication records. This translated into an average contribution of open-access sources of around 20% in recent years, where the spike in 2005 corresponds to one open-source publication of the two publications that year.

### 3.6. Authorship Networking Map

Table 7 shows the number of authors from the retrieved publications who obtained a minimum number of citations and publications for the sCO_2_-CSP topic. As can be observed, 1006 authors have published at least once on this topic, regardless of the number of citations received. This number falls significantly to 208 authors who have two sCO_2_-CSP-related publications and 10 citations.

**Figure 8:**
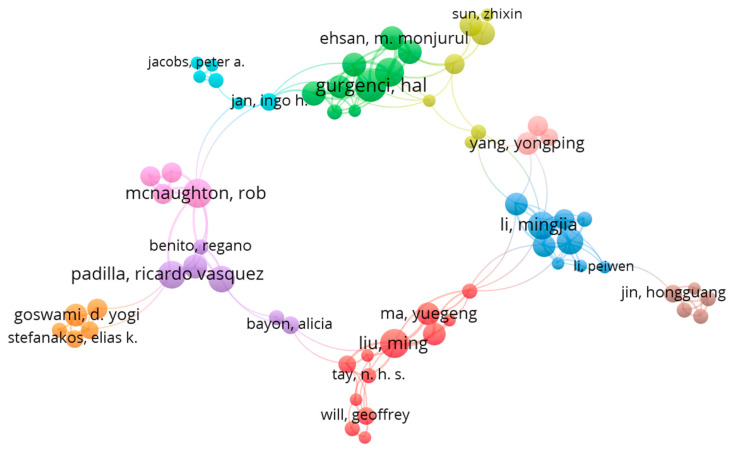
Network mapping of authors who met the minimum publication and citation criteria.

Figure 8 shows the authorship networking map for those authors fulfilling the two sCO_2_-CSP publications and 25 citations requirement. This criterion resulted in 152 authors; however, only 66 were connected in terms of collaborative publications that also met the minimum number of publications and citations criteria. For representation purposes, only connected authors are represented to explore their collaborations. A thesaurus was used to avoid duplications in authors’ names.

As can be noted in the map, authors are grouped under different clusters that indicate common collaboration. Furthermore, a repulsion representation scheme was chosen, which implies authors appearing closer to each other in the map have a closer relationship (in terms of collaborative publications) compared to those who appear more distant in the map. In addition, the size of the nodes is directly related to the number of authors’ publications. Table A1 in the Appendix A presents author clusters from Figure 8, indicating their affiliation.

Table 8 shows the number of institutions that have met the minimum number of citations and documents criteria attending to sCO_2_-CSP-related publications.

**Figure 9:**
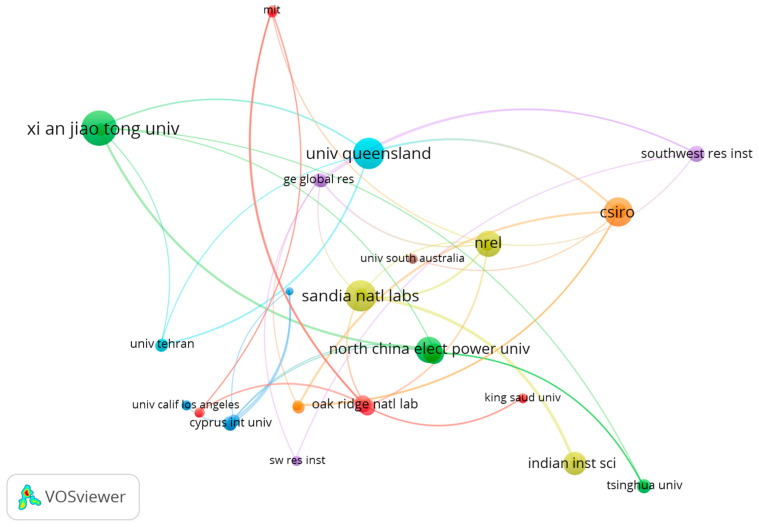
Authorship network mapping in terms of affiliation of authors who met the minimum publication and citation criteria.

Figure 9 shows the authorship networking map for the institutions affiliated with at least two co-authored publications related to the studied topic and a minimum of five citations. This criterion resulted in 107 institutions, but only 47 were connected and are represented on the map. The sizes of the nodes indicate the number of documents for each represented institution, the existence of connecting lines indicates collaborative publications among connected institutions, and the line thickness designates the number of collaborative publications. Table A2 in the Appendix A lists the organizations forming each cluster.

### 3.7. Publishing Sources Networking Map

Regarding publishing sources and their connections, Table 9 summarizes the number of sources relating to the minimum number of hosted publications and received citations. As shown, sCO_2_-CSP-related publications have been published in 105 different sources, but only 11 sources gather 10 or more publications on this topic, as also shown in Figure 6.

**Figure 10:**
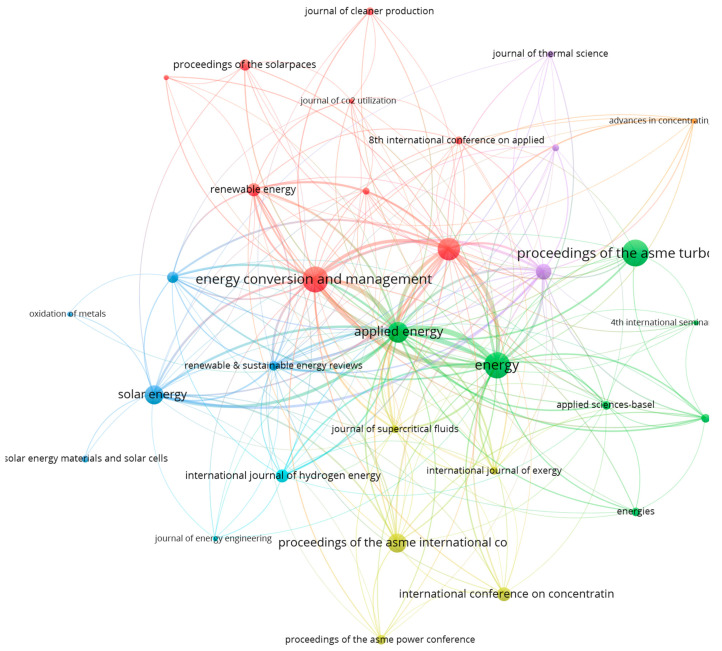
Publication sources’ connection networking map (at least 2 publications and 10 citations).

For representation purposes, Figure 10 shows network mapping connections among publishing sources having at least two publications on this topic and at least 10 citations. The sizes of the nodes indicate the number of documents of each journal, and the line thickness represents the strength in terms of citations between publications from connected journals. As shown, the journals are not all connected, which indicates that sCO_2_-CSP documents did not cite the other journal documents. A thesaurus was used to avoid duplications among different publishing sources, particularly for those from conference proceedings, which are grouped regardless of the year and edition. Table A3 presents the publishing sources of each cluster.

### 3.8. Bibliometric Summary Data

Table 10 summarizes the main bibliometric indicators presented in this section.

## 4. Discussion

In this section, technology trends for supercritical CO_2_ power cycles within concentrating solar power (CSP) applications are addressed, both from a semantic perspective (relating to the most common keywords extracted from publications) and the manner in which they are connected to the most recent research projects, both in Europe and in the United States.

### 4.1. Technology Trends

Text mining analysis was applied by extracting documents’ keywords from publication titles and abstracts, and those provided by authors from the retrieved sCO_2_-CSP publications. Table 11 shows the number of keywords relative to its number of occurrences. As shown, the 100 most common keywords appeared in at least five different publications.

**Figure 11:**
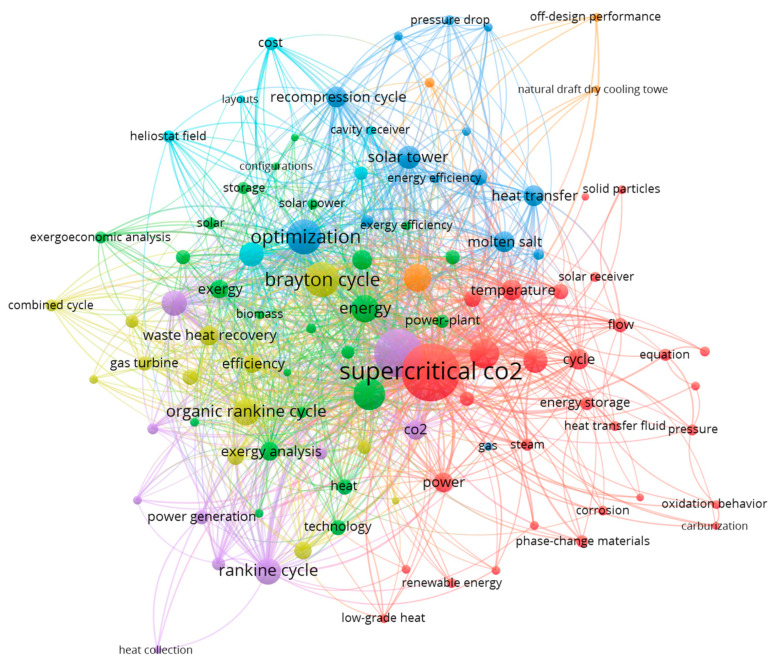
Keywords networking mapping with minimum of 5 occurrences.

Figure 11 shows how the 100 most common keywords within sCO_2_-CSP publications relate to each other. A similar repulsion and clustering scheme was followed in keywords representation, and can be summarized as follows:

- Keywords located in the center of the map are the most relevant and general within the retrieved publications because they are highly connected to other topics in the network (in this case “supercritical CO_2_”, “concentrating solar power”, “performance” and “system”).
- Keywords located in the peripheral area of the networking map are secondary within the topic of study because they are located far from the core of the network and with fewer connecting lines (as is the case of “heliostat field”, “combined cycle”, solid particles”, “phase-change materials”, “natural draft dry cooling tower” or “exergoeconomic analysis”).
- The size of nodes indicates the keyword relevance in terms of the number of occurrences; in this case, the most common are presented in Table 12.
- Keywords are grouped into clusters to indicate the frequency of their joint appearance in publications, denoting that they refer to similar research areas. In this study, keywords are organized in seven clusters dominated by “supercritical CO_2_”, “concentrating solar power”, “system”, “Brayton cycle”, “generation”, “optimization” and “designs” keywords.

Table 12 summarizes the most common keywords within the networking map related to the number of appearances and connections to other keywords in the network. The cluster number and the corresponding color is indicated for identification purposes within Figure 11.

**Figure 12:**
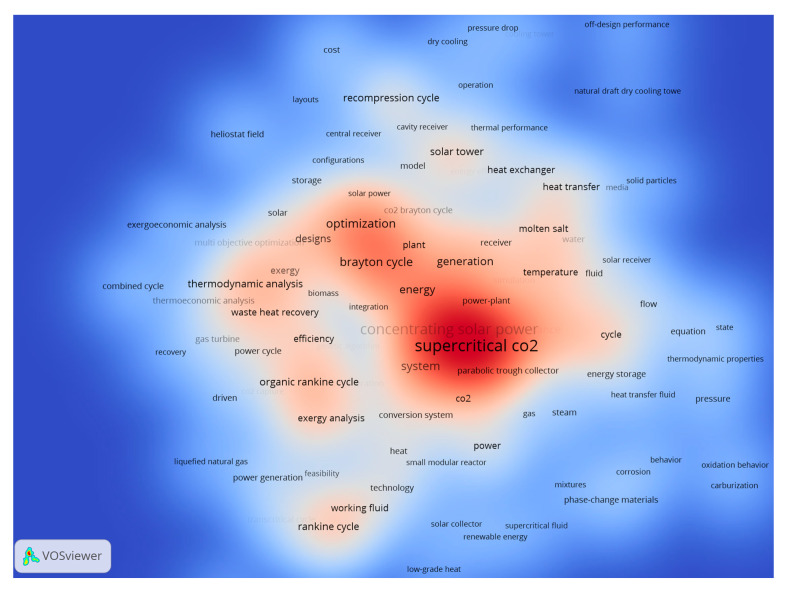
Density visualization of publication text mining analysis.

Figure 12 shows the density visualization map, which combines text mining extraction with the number of occurrences for each keyword. As shown, popular areas in the map are located around terms such as “optimization”, “thermodynamic analysis”, “efficiency”, “exergy analysis”, and “system”, which reflects the significance of thermodynamic studies for sCO_2_-CSP applications. However, most of those analyses relate to “performance analysis” and “multi objective optimization” according to the central areas of the map, whereas “off-design performance” studies remain in the periphery, indicating its lower relevance in terms of the number of publications. The incipient relevance of medium low-temperature applications within sCO_2_-CSP can also be observed as mild colored areas, including keywords such as “Rankine cycle”, “transcritical cycle”, “organic Rankine cycle”, “parabolic trough collector” and “waste heat recovery”. In addition, the growing relevance of “energy storage” can also be seen through common keywords of “thermal energy storage” and “phase-change materials”. Finally, “heat transfer” analysis and “heat exchanger” designs have gained relevance and are approaching the central area of the map.

### 4.2. Technology Prospectives: On-Going R&D Projects Combining CSP and sCO_2_ Applications 

Table 13 and Table 14, below, summarize all of the main ongoing R&D projects which explicitly refer to CSP and sCO_2_ systems in their objectives, in the EU and the USA, respectively. The tables present the name of the project, its general objective, and the project coordinator and participants, in addition to its duration, funding received, and funding agency. As of 2021, ongoing projects combining CSP and sCO_2_ can be divided into two groups: one focused on the system integration of sCO_2_ cycles with state-of-the-art CSP technologies; and the other focused on new systems, components, and materials at higher temperatures with lower maturity. Among the demonstration group of projects, SOLARSCO2OL and TESTBED can be highlighted, in EU and USA, respectively, which both aim at a MW-scale pilot to show the technical and economic viability of integrating a conventional CSP molten salt system with a novel sCO_2_ cycle, and are therefore limited to a turbine inlet temperature of approximately 565 °C. This is also the case of the pilot plant being developed by EDF in China, which involves the retrofitting of Shouhang’s 10 MW_e_ concentrated solar power plant that is operating at a maximum temperature of molten salt of 530 °C with a supercritical CO_2_ power cycle [72].

Other demonstration projects not included in the tables below, but of high relevance for CSP and sCO_2_, are the STEP project and Phase 3 of the US DOE Gen3 CSP program. The STEP project aims at demonstrating the technical viability of a 10 MW sCO_2_ cycle operating at 700 °C, at different configurations, with heat provided by natural gas. Phase 3 of the US DOE Gen3 CSP program, by comparison, focuses on demonstrating a new particle-based CSP system able to collect useful heat up to 900 °C, which can potentially enable high-temperature CSP-sCO_2_ systems in the future. In both the EU and the USA, particle-based systems appear to be the preferred path for future high-temperature CSP applications, at 700 °C or above. Considering that the maturity and commercial viability of such particle-based systems is yet to be proven, it can be estimated that, if sCO_2_ systems enter the CSP sector, then projects in the near term (i.e., up to 2030) will focus on using proven molten salt technology, thus indicating that most of the risk will relate to the sCO_2_ system itself.

As shown in the tables, most research projects involve significant optimization and system analysis activities, which reflects the significance of thermodynamic analysis for sCO_2_-CSP applications. It can also be observed that some recent research projects (CARBOSOLA and DESOLINATION) focus on the medium temperature applications of sCO_2_-CSP, as also shown in the text mining analysis in Figure 12. Also relevant are the growing number of research projects (SOLARSCO2OL SOLAR, and COMPASsCO2, and SETO 2018, SETO 2019, and SETO 2020) that are focusing on efficient heat exchanger designs, which is the key element connecting the solar field and the sCO_2_ power cycle; this corresponds to the identification in Figure 12 of popular topics such as “heat transfer” and “heat exchanger”. It may be argued that a direct relationship exists between popular areas of research that can be detected through literature text mining techniques, and the research project activities and pilot plant developments.

## 5. Conclusions

Research activities on supercritical CO_2_ (sCO_2_) for concentrating solar power (CSP) applications have gained significant attention in recent few years. This recent interest is based on high conversion efficiency predictions, which exceed 50% for the moderate temperature range, and the technology’s suitability for solar energy integration. This interest is also reflected in the large scientific bibliography (441 WOS indexed publications since 1993) and publicly funded research projects (24 projects in Europe and the United States since 2019). The main conclusions derived from the bibliometrics analysis conducted in this study are as follows:

- One-third of the existing sCO_2_ literature relates to solar energy applications;
- Rapid growth in sCO_2_ scientific publications has been observed, as 70% of the total number of documents were published after 2015 and 80% of citations were received after 2016;
- The most productive publishing countries during 2020 were China and Spain, which combined accounted for almost 50% of the total publications, and the top 10 most productive countries contributed a combined 86.5% of the total
- Considering the whole publishing timeframe, institutions from the United States, China, and Australia still dominate in terms of publishing and citations; this was confirmed by the high number of interactions among authors and institutions from these countries;
- Despite the large number of publishing sources (105), most documents were retrieved from 10 general energy-related sources, which are also the most connected in terms of citations;
- Regarding text-mining techniques applied to the indexed publications, the most common keywords referred to cycle optimization, system analysis, and performance studies; growing interest was observed for medium-low temperature applications through related keywords, such as Rankine cycle, organic Rankine cycle, and waste heat recovery;
- Areas of research related to heat exchanger design and energy storage solutions were detected through a density visualization map, which is consistent with the objectives of ongoing projects in Europe and the United States.

## Figures and Tables

**Table 1:** Related bibliometric analysis publications.

Author	Manuscript Title	Data Source	Year	Ref
Sultan, U. et al.	Qualitative assessment and global mapping of supercritical CO_2_ power cycle technology	Scopus and Web of Science	2021	[52]
Yu, A. et al.	Recent trends of supercritical CO_2_ Brayton cycle: Bibliometric analysis and research review	Scopus	2021	[53]
Reyes-Belmonte, M.A.	A Bibliometric Study on Integrated Solar Combined Cycles (ISCC), Trends and Future Based on Data Analytics Tools	Web of Science	2020	[54]
Calderon, A. et al.	Where is Thermal Energy Storage (TES) research going?—A bibliometric analysis	Web of Science	2020	[55]
David, T.M. et al.	Future research tendencies for solar energy management using a bibliometric analysis, 2000–2019	Scopus	2020	[56]
Saikia, K. et al.	A bibliometric analysis of trends in solar cooling technology	Web of Science	2019	[57]
Islam, M. et al.	A comprehensive review of state-of-the-art concentrating solar power (CSP) technologies: Current status and research trends	Web of Science	2018	[58]
Imran, M. et al.	Recent research trends in organic Rankine cycle technology: A bibliometric approach	Scopus	2018	[59]
Paulo, A.F. et al.	Solar energy technologies and open innovation: A study based on bibliometric and social network analysis Alex	Web of Science	2017	[60]
Du, H. et al.	A bibliographic analysis of recent solar energy literatures: The expansion and evolution of a research field	Web of Science	2014	[61]
Dong, B. et al.	A bibliometric analysis of solar power research from 1991 to 2010	Web of Science	2012	[62]

**Table 2:** Question query used for corpus data collection (1990–2020).

Question Query	Solar sCO_2_		sCO_2_	
	WOS	Scopus	WOS	Scopus
s-CO_2_ power cycle	113	124	357	409
Supercritical CO_2_ power cycle	373	341	1294	1295
Supercritical carbon dioxide power cycle	269	402	871	1441
sCO_2_ power cycle	29	152	113	421
Total corpus data	441	468	1509	1710

**Table 3:** Global publishing distribution for the topic of sCO_2_ for CSP applications.

Rank	1990–2019			2020		
	Country	Number of Publications	% of Publications	Country	Number of Publications	% of Publications
1	United States	117	21.7%	China	31	34.8%
2	China	113	20.9%	Spain	11	12.4%
3	Australia	55	10.2%	United States	9	10.1%
4	Spain	40	7.4%	Australia	5	5.6%
5	Japan	29	5.4%	United Kingdom	5	5.6%
6	India	25	4.6%	Iran	4	4.5%
7	Saudi Arabia	29	3.7%	Turkey	4	4.5%
8	South Korea	18	3.3%	Germany	3	3.4%
9	Italy	15	2.8%	India	3	3.4%
10	Iran	14	2.6%	Italy	2	2.2%
	Total		82.6%	Total		86.5%

**Table 4:** Most productive organizations in sCO_2_-CSP-related publications.

Rank	Organization	Country	Number of Publications	Number of Authors	Number of Citations	PC Ratio	h-Index
1	United States Department of Energy DOE	United States	54	128	1311	24.28	15
2	Xi’an Jiaotong University	China	32	70	1121	35.03	15
3	Doshisha University	Japan	26	23	1010	38.85	14
4	Sandia National Laboratory	United States	25	53	767	30.68	8
5	University of Queensland	Australia	25	39	348	13.92	10
6	Commonwealth Scientific Industrial Research Organisation CSIRO	Australia	22	39	643	29.23	13
7	North China Electric Power University	China	18	50	164	9.11	8
8	National Renewable Energy Laboratory NREL	United States	17	38	382	22.47	8
9	State University System of Florida	United States	16	24	511	31.94	7
10	Indian Institute of Science IISC Bangalore	India	15	27	243	16.20	8

**Table 5:** Most productive authors in sCO_2_-CSP.

Rank	Author	Institution	Country	Topic Documents	Topic Citations	PC Ratio	h-Index
1	Yamaguchi, H.	Doshisha University	Japan	25	985	39.4	14
2	Zhang, X.R.	Peking University	China	21	1018	48.48	15
3	Ho, C.K.	Sandia National Laboratory	United States	16	429	26.81	6
4	Gurgenci, H.	University of Queensland	Australia	13	93	7.15	5
5	Guan, Z.Q.	University of Queensland	Australia	10	95	9.5	5
6	Liu, M.	University of South Australia	Australia	10	150	15.0	6
7	McNaughton, R.	Commonwealth Scientific Industrial Research Organisation CSIRO	Australia	10	242	24.2	7
8	Sanchez, D.	University of Seville	Spain	10	339	33.9	5
9	Wang, J.F.	Xi’an Jiaotong University	China	10	463	46.3	7
10	Albrecht, K.J.	Sandia National Laboratory	United States	9	46	5.11	4

**Table 6:** Most cited publications in sCO_2_-CSP.

Rank	Publication	Publishing Source	Author	Institution	Country	Year	Citations	Citations/Year	Ref.
1	Review of Supercritical CO_2_ power cycle technology and current status of research and development	Nuclear Engineering and Technology	Ahn, Y. et al.	Korea Advanced Institute of Science and Technology	South Korea	2015	371	53.0	[1]
2	Review of high-temperature central receiver designs for concentrating solar power	Renewable and Sustainable Energy Reviews	Ho, C.K. and Iverson, B.D.	Sandia National Laboratory	United States	2014	344	43.0	[33]
3	Supercritical CO_2_ Brayton cycles for solar-thermal energy	Applied Energy	Iverson, B.D. et al.	Sandia National Laboratory	United States	2013	265	29.44	[20]
4	Thermodynamic Study of Advanced Supercritical Carbon Dioxide Power Cycles for Concentrating Solar Power Systems	Journal of Solar Energy Engineering	Turchi, C.S. et al.	National Renewable Energy Laboratory	United States	2013	224	24.89	[67]
5	Solar energy powered Rankine cycle using supercritical CO_2_	Applied Thermal Engineering	Yamaguchi, H. et al.	Doshisha University	Japan	2006	172	10.75	[68]
6	Supercritical carbon dioxide cycles for power generation: A review	Applied Energy	Crespi, F. et al.	University of Seville	Spain	2017	171	34.2	[2]
7	Parametric optimization design for supercritical CO_2_ power cycle using genetic algorithm and artificial neural network	Applied Energy	Wang, J. et al.	Xi’an Jiaotong University,	China	2010	147	12.25	[69]
8	Alternative cycles based on carbon dioxide for central receiver solar power plants	Applied Thermal Engineering	Chacartegui, R. et al.	University of Seville	Spain	2011	137	12.45	[70]
9	Exergetic analysis of supercritical CO_2_ Brayton cycles integrated with. solar central receivers	Applied Energy	Padilla, R.V. et al.	CSIRO	Australia	2015	136	19.43	[71]
10	Thermodynamic analysis and optimization of a molten salt solar power tower integrated with a recompression supercritical CO_2_ Brayton cycle based on integrated modeling	Energy Conversion and Management	Wang, K. and He, Y.	Xi’an Jiaotong University,	China	2017	134	26.80	[40]

**Table 7:** The number of authors meeting citation and publication criteria.

Minimum Number of Publications	Minimum Number of Citations						
	0	1	10	25	50	100	200
1	1006	859	489	281	163	84	44
2	278	263	208	152	113	63	38
5	66	66	66	62	53	35	26
10	9	9	9	9	9	6	5

**Table 8:** The number of institutions meeting citation and publication criteria.

Minimum Number of Publications	Minimum Number of Citations						
	0	1	10	25	50	100	200
1	300	263	166	101	67	34	18
2	114	110	94	65	53	27	17
5	31	31	31	29	27	18	15
10	11	11	11	11	11	10	9

**Table 9:** The number of publishing sources meeting citation and publication criteria.

Minimum Number of Publications	Minimum Number of Citations						
	0	1	10	25	50	100	200
1	105	87	51	27	21	15	10
2	40	37	32	20	17	13	9
5	18	18	18	15	15	12	9
10	11	11	11	11	11	9	8

**Table 10:** Main bibliometric indicators for sCO_2_-CSP WOS indexed publications.

Field	Value
Total number of publications	441
Total number of authors	1006
Total number of research institutions	300
Total number of publishing sources	105
Total number of countries	35
Sum of times cited	8855
Sum of times cited (without self-citations)	6693
Citing articles	4107
Citing articles (without self-citations)	3747
h-index	47
Average citations per item	20.08

**Table 11:** The minimum number of occurrences of a keyword.

Minimum Number of Occurrences	Number of Keywords
1	1259
2	302
5	103
7	81
10	62
20	30
50	10

**Table 12:** Most common keyword ranking.

Ranking	Keyword	Number of Appearances	Number of Connections	Cluster Identification
1	Supercritical CO_2_	250	101	#1 (red)
2	Concentrating Solar Power	174	101	#5 (purple)
3	Optimization	93	93	#3 (blue)
4	System	75	87	#2 (green)
5	Performance	69	90	#1 (red)
6	Brayton cycle	64	86	#4 (yellow)
7	Generation	59	85	#7 (orange)
8	Energy	58	81	#2 (green)
9	Organic Rankine Cycle	51	75	#4 (yellow)
10	Thermodynamic analysis	49	74	#5 (purple)
11	Thermal Energy Storage	45	74	#1 (red)
12	Designs	44	74	#6 (cyan)
13	Solar Tower	42	76	#3 (blue)
14	Recompression cycle	35	65	#3 (blue)
15	CO_2_ Brayton cycle	34	63	#3 (blue)

**Table 13:** Selected ongoing projects in the EU specifically referring to CSP and sCO_2_ in their objectives (as of July 2021) [73,74,75,76,77,78].

Project	General Objectives	Project Coordinator and Partners	Project Duration and Received Grant
ACES2030-CM Concentrated solar thermal energy in the transport sector and the production of heat and electricity	The collaborative structure in ACES2030 promotes synergy between facilities and laboratories around solar thermal technology in support of the industry’s R&D activities, with the ambition of being the seeds of a future network of unique infrastructures in the Community of Madrid. In particular relation to sCO_2_, the project aims to develop technologies for next-generation concentrated solar thermal power plants that are efficient, operational, and competitive in a scenario of increasing electrification of society. This objective is aligned with the recent priorities set out in the US Department of Energy’s Gen3 CSP program, and primarily with pressurized gas technology (sCO_2_).	IMDEA Energia, CIEMAT, Universidad Carlos III, CSIC, UNED, Universidad Rey Juan Carlos, Universidad Politécnica Madrid, Abengoa Energia, Empresarios Agrupados, Grupo Cobra, Protermosolar, Repsol, Rioglass Solar	2019–2023 EUR 1.0 M Comunidad de Madrid, Spain (S2018/EMT-4319) co-funded with structural funds of the European Union
SCARABEUS Supercritical CARbon dioxide/Alternative fluids Blends for Efficiency Upgrade of Solar power plants	The project aims to demonstrate that the application of supercritical CO_2_ blends to CSP plants. There are two main areas of research in this project: the first is the identification of the optimal additives, which would reduce the size and increase the efficiency of the power block. The second is the development of tailored heat exchanger designs, particularly for the air-cooled condenser, to operate with the innovative fluid, because these are key enabling components for the proposed technology. The project will demonstrate the innovative fluid and newly developed heat exchangers at a relevant scale (300 kWth) for 300 h in a CSP-like operating environment (700 °C).	Politecnino di Milano, TU Wien, Universidad de Sevilla, City University of London, Universita’ degli Studi di Brescia, Kelvion Thermal Solution, Baker Hughes, Abengoa, Quantis	1 April 2019–31 March 2023 EUR 5.0 M European Commission (GA 814985)
CARBOSOLA supercritical carbon dioxide (sCO_2_) as an alternative working fluid for downstream processes and solar-thermal applications—Design methods for sCO_2_ power plant technology	The CARBOSOLA project is intended to be the entry into the development of sCO_2_ technology in Germany. The main goal of the industrial partner Siemens is the conceptual design of a demonstrator with which the validation of the sCO_2_ technology is performed. The core of the project is the component and system design of a technology demonstrator for the use of secondary heat and the development of the theoretical and experimental methods needed for further technology development to commercial maturity.The sCO_2_ technology will first be compared with conventional technologies in the areas of recuperation of waste heat (downstream processes for gas turbine plants) and solar thermal power plant technology (CSP) and subjected to a technical-economic evaluation	Technische Universität Dresden, Helmholtz-Zentrum Dresden-Rossendorf, DLR, SIEMENS AG	1 October 2019–30 September 2022 EUR 0.4 M Ministry for Economic Affairs and Energy (BMWi), Germany (GA 03EE5001B)
SOLARSCO2OL SOLAR based supercritical Carbon Oxide Operating Low-cost plants	SOLARSCO2OL aims at developing an innovative, economically viable, and replicable supercritical CO_2_ (sCO_2_) power block for demonstrating the use of sCO_2_ cycles as a potential key technology to increase the flexibility of concentrated solar power (CSP) plants. This will reduce their levelized cost of electricity (LCOE) to values below 10 c€/kWh_e_ in Europe and promote an innovative power plant cycle layout not requiring water. The innovative SOLARSCO2OL plant layout, coupled with fast-reactive electric heaters and efficient heat exchangers (HEXs), will enable the operation and design of novel integrated CSP plant layouts.	RINA Consulting, Kungliga Tekniska Högskolan (KTH), Masen, Ikerlan, Universita Degli Studi Di Genova, CERTH, Magtel, Franco Tosi Meccanica, ESTELA, MAS, Lointek, Baker Hughes, Seico, Abengoa, OCMI OTG	1 October 2020–30 September 2024 EUR 10.0 M European Commission (GA 952953)
COMPASsCO_2_ Components’ and Materials’ Performance for Advanced Solar Supercritical CO_2_ Power plants	The COMPASsCO_2_ project aims at integrating solar energy into sCO_2_ Brayton cycles for electricity production. The project will design, test, and model tailored particle-alloy combinations able to face the extreme operating conditions regarding temperature, pressure, abrasion, oxidation, and corrosion during the plant lifetime. Testing of the particle-sCO_2_ heat exchanger will validate the innovative materials developed.	DLR, CIEMAT, John Cockerill, Research Center REZ, Dechema Research Institute, Julich Research Center, OCAS, Observatoire Mediterraneen De L’energie, Saint-Gobain, Sugimat, University of Birmingham, Teknologian Tutkimuskeskus (VTT)	1 November 2020–31 October 2024 EUR 6.0 M European Commission (GA 958418)
DESOLINATION DEmonstration of concentrated SOLar power coupled wIth advaNced desAlinaTion system in the gulf regION	The DESOLINATION project aims to efficiently couple the low-grade wasted heat of two different CSP cycles to an innovative desalination system based on forwarding osmosis. The demonstration in Saudi Arabia already hosts a 100 kWe air Bryton cycle that will be coupled with the innovative forward osmosis desalination system developed in DESOLINATION. Moreover, to consider the future and most efficient cycles, a 1 MWe CO_2_ blended power cycle will be installed onsite and demonstrated alongside the existing power plant. DESOLINATION will thus provide solutions to be integrated into existing CSP plants across the region, and an innovative more efficient coupling with a tailored power cycle for more efficient and cost-effective new CSP plants based on CO_2_ blends.Through the developments of the CSP+D system and its demonstration in a real environment, DESOLINATION will foster the use of solar energy for desalination in the EU, in the GCC countries, and the rest of the world.	Polytechnic University of Milan, Lund University, Protarget, Baker Hughes, ACS Cobra, Fraunhofer ISE, Aalborg CSP, Cranfield University, Fundacion Tekniker, Lappeenranta University of Technology (LUT), University of Brescia, Eindhoven University of Technology, Temisth, University of Maribor, Luleå University of Technology, Euroquality, King Saud University, University of Bahrain, German University of Technology in Oman	1 June 2021–31 May 2025 EUR 10.0 M European Commission (GA 101022686)

**Table 14:** Selected ongoing R&D projects in the USA specifically referring to CSP and sCO_2_ in their objectives (as of July 2021) [79,80,81,82,83,84,85,86,87,88,89,90,91,92,93,94,95].

Project	General Objectives	Project Coordinator and Partners	Project Duration and Received Grant
SETO 2018 Mechanically, Thermally, and Chemically Robust High-Temperature Ceramic Composites	To evaluate the corrosion and heat resistance of new ceramic-metal composite materials for use in components in concentrating solar-thermal power (CSP) plants. Objectives: Develop composites stiffer and stronger than nickel-based superalloys at 550–750 °C. Test the heat and corrosion resistance of these composite working with air, sCO_2_ and chloride salts Evaluate less expensive methods of manufacturing components from these materials.	Purdue University	2019–2021 USD 0.4 M US DOE
SETO 2018 740H Diffusion Bonded Compact Heat Exchanger for High Temperature and Pressure Applications	This project team is developing new manufacturing techniques for an advanced alloy, Inconel 740H, which has extremely high strength at the temperatures required for next-generation CSP plants. Specific Objectives: Develop manufacturing processes using iterative testing of different approaches to address challenges involved in using 740H Improve etching a diffusion bonding techniques for 740H Test a prototype heat exchanger made of 740H and produced using industry-standard manufacturing techniques, at a 100 kW scale	CompRex LLC, Special Metals, University of Wisconsin-Madison, Advanced Vacuum Systems	2019–2021 USD 1.2 M US DOE
SETO 2018 Additively Manufacturing Recuperators via Direct Metal Laser Melting and Binder Jet Technology	Develop additive manufacturing processes for the heat exchangers in sCO_2_ cycles. Use binder jet printing to enable new heat exchanger geometries (3D channels, curved features) Evaluate the new process and determine if it’s capable of producing CSP compatible power cycles that cost 900 USD/kW and produce energy at 0.05 USD/kWh_e_ Perform mechanical tests to ensure that the resulting heat exchangers can withstand the high operating temperatures and pressures Create a risk reduction plan for scaling the heat exchanger design from lab-scale to a full-scale, including, a modular design	General Electric	2019–2021 USD 1.4 M US DOE
SETO 2018 Reduced Levelized Cost of Energy in CSP Through Utilizing Process Gas Lubricated Bearings in Oil-Free Drivetrains	De-risk a novel bearing design for the turbines used in concentrating solar-thermal power (CSP) plants with sCO_2_ power cycles. Replace existing oil lubrication with gas-bearing lubrication technology, to increase plant efficiency, reduce maintenance costs, and reduce the manufacturing costs of power blocks. Objectives: Perform mechanical tests and simulate rotor tests Perform techno-economic analysis to determine if the design can achieve a 50% efficient power cycle to lower costs to 0.05 USD/kWh_e_	General Electric	2019–2021 USD 2.4 M US DOE
SETO 2018 Development of a High-Efficiency Hybrid Dry Cooler System for sCO_2_ Power Cycles in CSP Applications	Develop a compact dry cooling heat exchanger for supercritical carbon dioxide (sCO_2_) power cycles in CSP plants. Objectives: Create and optimize a dry cooling heat exchanger with microchannels on the sCO_2_ side and a geometry that uses plates and finned chambers on the airside. Test the dry cooling system at the megawatt scale with a sCO_2_ test loop, to determine the reliability of the fabrication method, and validate performance. The improvements could increase the cooling efficiency to 90%, reduce the cooler cost from 168 USD/kW to 95 USD/kW and reduce cooling power consumption by 14%.	Southwest Research Institute	2019–2021 USD 1.9 M US DOE
SETO 2018 High-Temperature Dry-Gas Seal Development and Testing for sCO_2_ Power Cycle Turbomachinery	This project will develop a high-temperature dry gas seal (DGS) by replacing the temperature-sensitive elements with more durable components, enabling the DGS to reach operating temperatures over 500 °C and enable higher efficiency levels. Because the DGS design would also be significantly smaller in size, the DGS would reduce the complexity of the sCO_2_ turbine design, helping to increase operational reliability and improve turbine efficiency. Specific objectives Replace the polymers in the dry gas seal with materials that carry the same properties but can withstand higher temperatures Test and validate materials in a dry gas seal package at a temperature of 500 °C By simplifying the turbine’s heat-shielding requirements, the new technology should improve the efficiency of sCO_2_ power turbines by up to 4%.	Southwest Research Institute	2019–2021 USD 2.0 M US DOE
SETO 2018 Additively-Manufactured Molten Salt-to-Supercritical Carbon Dioxide Heat Exchanger	Develop an additively manufactured, nickel superalloy primary heat exchanger (PHX) for advanced molten salt concentrated solar-thermal power (CSP) systems. The PHX will be made using nickel superalloys and laser powder bed 3D printing, resulting in a compact design that is durable under cyclic operation at high temperatures and pressures in a corrosive salt environment. Objectives: Characterize and test different alloy powders both in conditions representative of Gen 3 CSP systems—720 °C and supercritical carbon dioxide pressures of 200 bar—and at conditions relevant to current commercial systems—molten nitrate salt at temperatures up to 550 °C. Validate a thermal model that can predict performance in a chloride salt environment Develop a 20-kilowatt design to test the mechanical integrity of the fabricated PHX.	University of California Davis	2019–2021 USD 2.2 M US DOE
SETO 2018 Narrow-Channel, Fluidized Beds for Effective Particle Thermal Energy Transport and Storage	Develop and test narrow-channel, counterflow fluidized bed receiver and heat exchanger designs. These will be used to analyze flow conditions and improve heat transfer rates in the receiver and heat exchanger. The team will then use these insights to test a modular panel for an indirect particle receiver and/or particle to a supercritical carbon dioxide power cycle heat exchanger. Objectives: Achieve heat exchange efficiency higher than 90% at 700 °C inlet temperature Deliver detailed multiphase flow modelling tools to assess how receiver and heat exchanger designs can meet receiver cost targets of 150 USD/kWh_th_ and thermal-energy system targets of 15 USD/kWh_th_	Colorado School of Mines, Sandia National Laboratories, Carbo Ceramics	2019–2021 USD 1.9 M US DOE
SETO 2019 Economic Weekly and Seasonal Thermochemical and Chemical Energy Storage for Advanced Power Cycles	Integrate multiple thermochemical energy storage components into a concentrating solar-thermal power (CSP) design so that a plant can have multiple storage durations, including daily and long-term. Objectives: Design TES for sCO_2_ power loop integration Conduct techno-economic analyses to improve CSP system design and operation for guaranteed year-round energy dispatchability.	Arizona State University, Oregon State University, Sandia National Laboratories, Siemens, Southwest Research Institute	2020–2022 USD 3.3 M US DOE
SETO 2019 Creep and Fatigue Characterization of High-Strength Nickel Alloys Thin Sections in Advanced CO_2_ Heat Exchangers	Examine creep behavior in thin-sheet nickel alloys 740H and 282, to see whether they can improve the lifetime of supercritical carbon dioxide (CO_2_) heat exchangers in high-temperature concentrating solar-thermal power plants. Objectives: Provide information about structural characteristics in metals used to build heat exchangers Determine the optimal thickness of these components Heat exchanger performance modelling Basic materials research and fabrication of test specimens for characterization Experimental design and bench-scale laboratory experiments	Brayton Energy, Oak Ridge National Laboratory	2020–2022 USD 0.7 M US DOE
SETO 2019 Advanced Compressors for CO_2_-Based Power Cycles and Energy Storage Systems	Develop a large-scale, low-cost, single-shaft compressor for supercritical carbon dioxide (sCO_2_) power cycles and energy storage systems to improve the performance of concentrating solar-thermal power systems.	Echogen Power System, University of Notre Dame	2020–2022 USD 4.4 M US DOE
SETO 2019 Near-Net-Shape Hot Isostatic Press Manufacturing Modality for sCO_2_ CSP Capital Cost Reduction	Fabricate advanced supercritical carbon dioxide (sCO_2_) power cycle structures for CSP plants from metal powders by using powder metallurgy, near-net-shape (NNS) hotisostatic pressed (HIP) technology. Objectives: A turbine nozzle ring, turbine case, cylindrical structure, and dual alloy pipe would be fabricated as a demonstration of the technology’s viability Activities to be performed would include material characterization (e.g., alloy powder assessment), data collection, component design, component fabrication (e.g., prototype nozzle ring, casing, and dual-alloy pipe), validation testing (e.g., microstructural analysis), and cost modelling.	General Electric, Synerthec	2020–2022 USD 2.5 M US DOE
SETO 2019 Vertically Aligned Carbon Nanotube Arrays as Novel, Self-Lubricating, High-Efficiency Brush Seal for CSP Turbomachinery	Develop a new scalable seal brush on a flexible base that will improve the seal’s efficiency and durability. The seal will be made of a vertically aligned carbon nanotube array and use a chemical vapor deposition process without a catalyst. The main aim is to improve turbine efficiency and reduce the manufacturing cost by at least half.	Oak Ridge National Laboratory	2020–2022 USD 1.4 M US DOE
SETO 2019 Oxidation-Resistant, Thermomechanically Robust Ceramic-Composite Heat Exchangers	Develop cost-efficient ceramic-composite primary heat exchangers that are highly resistant to corrosion by supercritical carbon dioxide and molten salt and will not deform or fracture at temperatures as high as 800 °C. Objectives: Developed HEx to be resistant to corrosion, creep, fracture, and thermal cycling when transferring heat from high-temperature molten salt to supercritical carbon dioxide-based fluid Test the Hex under relevant working conditions	Purdue University, Massachusetts Institute of Technology, TharEnergy	2020–2023 USD 3.5 M US DOE
SETO 2020 Integrated TESTBED	Develop, build, and operate a sCO_2_ power cycle integrated with thermal energy storage at temperatures in the range of 550 to 630 °C. Objectives: Develop, build, and operate a supercritical carbon dioxide (sCO_2_) power cycle integrated with thermal energy storage, heated by a concentrated solar thermal energy supplied by a newly built heliostat field. Operate at a TIT of 600 °C	Heliogen Inc.	2021–2024 USD 39.0 M US DOE
SETO 2020 Small Innovative Projects in Solar (SIPS)—Enhancing Particle-to-sCO_2_ Heat Exchanger Effectiveness Through Novel High-Porosity Metallic Foams	This project aims to increase the effectiveness of particle-to supercritical carbon dioxide (sCO_2_) heat exchangers by packing the particle-side channels with high-porosity cellular structures. The approach includes metal additive manufacturing of small length-scale fibers with complex three-dimensional interconnections. Objectives: Increase the interstitial heat-transfer coefficient between moving particles and metallic fibers, and the effective thermal conductivity of particle channel. Test the Hex design at Sandia test rig Scaling up of the technology	Mississippi State University, Sandia National Laboratories, National Renewable Energy Laboratory	2021–2022 USD 0.3 M US DOE
SETO 2020 Small Innovative Projects in Solar (SIPS)—Enabling Robust Compressor Operation under Various sCO_2_ Conditions at Compressor Inlet	This project team will study how supercritical carbon dioxide (sCO_2_) flows in a compressor cascade in a concentrating solar-thermal power system. Objectives: Develop a new design methodology for the compressor’s leading-edge suction surface so that the compressor can work well over a range of ambient conditions, without problems caused by condensation Identify and quantify condensation at the compressor’s leading edge, and characterize detailed sCO_2_ flows within the compressor	University of Central Florida, CRAFT Tech	2021–2022 0.3 M$ US DOE

## Appendix A

**Table A1:** Authors’ distribution by cluster (from Figure 8).

Author	Affiliation	Author	Affiliation
Cluster #1 (red)		Cluster #2 (green)	
Bell, S.	Queensland University of Technology	Duniam, S.	University of Queensland
Belusko, M.	University of South Australia	Ehsan, M.	University of Queensland
Bruno, F.	University of South Australia	Guan, Z.	University of Queensland
Liu, J.	Xi’an Jiaotong University	Gurgenci, H.	University of Queensland
Liu, M.	University of South Australia	Hooman, K.	University of Queensland
Ma, Y.	Xi’an Jiaotong University	Klimenko, A.	University of Queensland
Sarvghad, M.	Queensland University of Technology	Sun, Y.	North China Electric Power University
Steinberg, T.A.	Queensland University of Technology	Veeraragavan, A.	University of Queensland
Tay, N.H.S.	University of South Australia	Wang, J.	Xi’an Jiaotong University
Will, G.	Queensland University of Technology		
Yan, J.	Xi’an Jiaotong University		
Zhang, X.	Peking University		
Cluster #3 (red)		Cluster #4 (red)	
Guo, J.	Xi’an Jiaotong University	Dai, Y.	Xi’an Jiaotong University
He, Y.	Xi’an Jiaotong University	Li, X.	North China Electric Power University
Li, M.	Xi’an Jiaotong University	Liu, C.	North China Electric Power University
Li, P.	University of Arizona	Sun, Z.	Xi’an Jiaotong University
Liu, Z.	Xi’an Jiaotong University	Wang, J.F.	Xi’an Jiaotong University
Qiu, Y.	Xi’an Jiaotong University	Wang, X.	Chinese Academy of Sciences
Wang, K.	Xi’an Jiaotong University	Xu, X.	University of Arizona
Xu, J.	North China Electric Power University		
Zhu, H.	Xi’an Jiaotong University		
Cluster #5 (red)		Cluster #6 (red)	
Bayon, A.	CSIRO	Jacobs, P.	The University of Queensland
Benito, R.	CSIRO	Jan, I.	The University of Queensland
De la calle, A.	CSIRO	Kearney, M.	The University of Queensland
Padilla, R.V.	CSIRO	Miller, S.	CSIRO
Stein, W.	CSIRO	Rowlands, A.	The University of Queensland
Too, Y.S.	CSIRO	Singh, R.	The University of Queensland
Cluster #7 (red)		Cluster #8 (red)	
Besarati, S.	University of South Florida	Bai, Z.	Chinese Academy of Sciences
Chen, H.	Suzhou Adv Mat Res Inst	Jin, H.	Chinese Academy of Sciences
Goswami, D.	University of South Florida	Lei, J.	North China Electric Power University
Rahman, M.	University of South Florida	Liu, Q.	Chinese Academy of Sciences
Stefanakos, E.	University of South Florida	Wang, X.	Chinese Academy of Sciences
Cluster #9 (red)		Cluster #10 (red)	
Abbas, A.	University of Sydney	Li, X.	Chongqing University
Mcnaughton, R.	CSIRO	Xu, C.	North China Electric Power University
Milani, D.	University of Sydney	Yang, Y.	North China Electric Power University
Minh, T.	University of Sydney		

**Table A2:** Organization distribution by cluster (from Figure 9).

Organization	Country	Organization	Country
Cluster #1 (red)		Cluster #2 (green)	
Georgia Inst Technology	United States	Beijing University	China
Hunan University	China	Chinese Academy of Sciences	China
King Saud University	Saudi Arabia	North China Electric Power University	China
MIT	United States	Technical University Berlin	Germany
Oak Ridge National Lab	United States	Tsinghua University	China
Purdue University	United States	University of Arizona	United States
Saudi Electricity Co	Saudi Arabia	University of Chinese Academy of Sciences	China
University of Wisconsin	United States	Xi’an Jiaotong University	China
Cluster #3 (blue)		Cluster #4 (yellow)	
Cyprus Int Univ	Cyprus	Colorado School of Mines	United States
Mirpur University	Pakistan	Indian Institute of Sciences	India
Must	Pakistan	NREL	United States
Natl Univ Sci & Tech	Pakistan	Sandia Natl Labs	United States
University of California	United States	Universidad Carlos III	Spain
Virginia Tech	United States	University of Western Australia	Australia
Zhejiang University	China		
Cluster #5 (purple)		Cluster #6 (light blue)	
GE Global Res	United States	Henan University	China
Hanwha Techwin	South Korea	Shahrood University	Iran
Montana State University	United States	University of Queensland	Australia
Southwest Res Inst	United States	University of Tehran	Iran
SW Res Inst	United States	Wuhan University	China
US DOE	United States		
Cluster #7 (purple)		Cluster #8 (light blue)	
Australian National University	Australia	Queensland University	Australia
CSIRO	Australia	University of South Australia	Australia
Southern Cross University	Australia		
University of Sydney	Australia		
Univ Tech Federico Santa Maria	Chile		

**Table A3:** Publishing sources’ distribution by cluster (from Figure 10).

Publishing Source	Publishing Source
Cluster #1 (red)	Cluster #2 (green)
8th International Conference on Applied Energy	4th International Seminar on ORC power systems
Applied Thermal Engineering	Applied Energy
Energy Conversion and Management	Applied Sciences
International Journal of Heat and Mass transfer	Energies
Journal of cleaner production	Energy
Journal of energy resources technology—Transactions of the ASME	Journal of Engineering for Gas Turbines and Power—Transactions of the ASME
Proceedings of the SolarPaces	Proceedings of the ASME Turbo Expo
Renewable Energy	
Cluster #3 (blue)	Cluster #4 (yellow)
International Journal of Energy Research	International Conference on Concentrating Solar Power and Chemical
Oxidation of Metals	International Journal of Exergy
Renewable & Sustainable Energy Reviews	Journal of Supercritical fluids
Solar Energy	Proceedings of the ASME International Conference on Energy
Solar Energy Materials and Solar Cells	Proceedings of the ASME Power Conference
Cluster #5 (purple)	Cluster #6 (light blue)
Journal of Solar Energy Engineering—Transactions of the ASME	International Journal of Hydrogen Energy
Journal of Thermal Science	Journal of Energy Engineering
Processes	
Cluster #7 (orange)	
Advances in Concentrating Solar Thermal	
